# Identifying informal leaders among medical residents as a basis for educational interventions

**DOI:** 10.1186/s12909-026-08918-0

**Published:** 2026-02-28

**Authors:** Patrick Ferreira da Silva, Tarcisio Abreu Saurin, Flavio Sanson Fogliatto, José Miguel Dora, Dimitris Rucks Varvaki Rados

**Affiliations:** 1 Industrial Engineering Department, Federal University of Rio Grande Do Sul (UFRGS), Porto Alegre, Brazil; 2 Department of Internal Medicine, School of Medicine, Federal University of Rio Grande Do Sul (UFRGS), Porto Alegre, Brazil

**Keywords:** Informal leadership, Medical residents, Internal medicine, Social network analysis, Educational interventions

## Abstract

**Background:**

Leadership skills training is not usually integrated into the curriculum of medical residents, contributing to variation in skills development. Hence, identifying the manifestations of residents’ informal leadership can guide educational interventions. It is unclear how theories of formal leadership can support the identification of informal leaders.

**Purpose:**

To propose an approach for identifying the manifestations of informal leadership among medical residents, using this information for proposing interventions.

**Methods:**

A social network analysis survey was conducted including 68 physicians (22 preceptors and 46 residents) from an internal medicine service of a university hospital using data on advice-seeking relationships, self-assessments of four leadership styles (transformational, relational, adaptive, resilient), and preceptors’ evaluations of the styles’ importance. For each participant, a leadership score was calculated by integrating four network metrics (in-degree, betweenness, in-closeness, and out-closeness) that were proxies of the leadership styles. This score reflected “leadership-as-done” (LAD). The responses to the self-assessment questions reflected “leadership-as-imagined” (LAI). The scores for both LAD and LAI were weighted by the importance of leadership styles. A scatterplot placed each participant by LAI and LAD scores, partitioned into four groups: established, humble, aspirational, and latent leaders. A residents-only network was additionally examined to compare group assignment when preceptors were excluded.

**Results:**

Considering the full network with residents and preceptors, which more accurately represents the service complexity, 15.2% of the residents were established leaders, 23.9% were humble leaders, 21.7% were aspirational leaders, and 39.1% were latent leaders. Established and humble leaders stand out as informal leaders. The former regard their skills well developed and play central roles in the network; the latter do not consider their skills well developed, while still being central.

**Conclusions:**

Educational interventions should focus on offering advanced training for established leaders, enhancing the leadership skills of humble leaders that match their real demand for advice, giving opportunities for increasing the network centrality of aspirational leaders, and providing supervised leadership experiences for latent leaders.

**Supplementary Information:**

The online version contains supplementary material available at 10.1186/s12909-026-08918-0.

## Introduction

Leadership in medical residency programs plays a crucial role in professional identity, team dynamics, and patient safety [1–3]. Professional identity develops as residents come to see themselves as legitimate practitioners, shaped by participation in clinical work and iterative learning cycles [4]. Leadership identity is a dimension of professional identity formation, emerging when residents develop awareness of their informal leadership role and skills through practice in specific situations [5]. In fact, leadership in clinical environments usually arises informally, especially among medical residents (MRs) who undertake critical responsibilities in the absence of senior physicians and lead junior staff [6–8]. Even though these informal leadership roles are important, residency programs often leave leadership-related skills (e.g., communication, conflict resolution, and decision-making) to the “hidden curriculum”, which is not explicitly taught, labeled, or assessed within the formal syllabus [7, 9, 10]. This problem is compounded by the inconsistent supervision MRs receive from preceptors [7, 11]. Leadership skills training consists of educational approaches designed to develop observable leadership behaviors in clinical work through guided practice and feedback [11].

Theories of formal leadership offer a point of departure for understanding informal leadership, as both types usually coexist in the same environment and address similar healthcare challenges [12]. However, previous studies do not clarify how these theories can be applied to identify informal leaders [13, 14]. This gap is important since recognizing informal leaders among MRs enables targeted interventions to enhance leadership skills. Such skills are valuable for all MRs, as they may be required to lead at various points in their daily clinical work, regardless of whether they are considered informal leaders. Leadership will also be needed in their professional careers beyond residency [15–17].

Moreover, previous studies of MR leadership, either formal or informal, do not account for the gap between self-perceived leadership and leadership displayed in practice. This misalignment resembles the gap between work-as-imagined and work-as-done. Work-as-Imagined (WAI) refers to how tasks, roles and responsibilities are prescribed in guidelines and plans, whereas Work-as-Done (WAD) captures the adaptations and interpersonal dynamics that occur under real-world constraints and uncertainty [18–20]. The gap between WAI and WAD has been extensively studied in the health services literature, and it is attributed to the complexity of healthcare that implies a high level of inevitable uncertainty and variability that cannot be fully anticipated by prescriptions [18–20].

Based on this lens, leadership in medical residency can be understood as a dual construct comprising Leadership-as-Imagined (LAI) and Leadership-as-Done (LAD). In line with leadership identity [14], LAI is an indicator of leadership “claims”, whereas LAD reflects the extent to which these claims are “granted” by colleagues through their advice-seeking ties. Consequently, leadership may be enacted without being explicitly self-identified, which allows to interpret LAI–LAD misalignment as a gap between leadership practice and leadership identity. LAI in residency training is conceptualized as a set of competencies and behaviors that physicians are expected to demonstrate such as clear communication, conflict management, team coordination and the ability to motivate colleagues [21, 22]. This normative view is formalized through competency frameworks, leadership curricula and assessment tools, and it also manifests when residents rate themselves on leadership scales derived from formal leadership theories [7, 15].

Therefore, it is also necessary to investigate the actual influence patterns (LAD) in the daily network of professional relationships. To this end, this study uses Social Network Analysis (SNA), which reveals the influence that shapes collective work in practice [23]. SNA enables the visualization and examination of connections within different types of networks, from information science to human resources, and is extensively applied in healthcare to improve patient care [23–26]. Grounded in graph and network theory, SNA examines behavioral patterns, social influence, and teamwork by analyzing relationships between network individuals [27, 28]. It is typically implemented through questionnaire surveys in which participants indicate individuals they rely on for information or advice [29, 30]. These surveys may also include questions about the participant’s profile (e.g., alignment with certain leadership styles), which can be analyzed alongside responses related to the peer-consultation relationships [30].

Building on this approach, this paper focuses on internal medicine residents, for whom leadership skills are crucial to supervising junior staff, coordinating patient care, and engaging in quality improvement initiatives [31–33]. This investigation draws on the core ideas of transformational, relational, adaptive, and resilient leadership theories, which align with the highly complex internal medicine context [34–37]. Transformational leadership highlights a leader’s ability to inspire and motivate members toward a shared vision, fostering innovation and continuous improvement [34, 35]. These are essential abilities for internal medicine residents who guide clinical teams, promote open discussions, and build collective consensus [22]. Relational leadership focuses on collaboration and engagement among team members, making it well suited to the multidisciplinary care teams often led by internal medicine residents [36]. Resilient leadership emphasizes emotional stability in the face of challenging or unexpected clinical scenarios, which are part of residents’ daily work [38, 39]. Adaptive leadership addresses coping with ambiguity and uncertainty when no single standardized solution exists, a situation frequently encountered in internal medicine [40]. Both LAI and LAD in internal medicine residency might be regarded as emergent properties of the interactions between these four leadership styles. A leadership style corresponds to the manifestation of the core ideas of a leadership theory in practice.

Considering this background, the research question addressed by this study is stated as follows: how can formal leadership theories be used for the identification of informal leaders among medical residents, supporting targeted educational interventions? The research question was empirically examined in a large university hospital, allowing the development of a novel SNA-based approach for identifying informal leaders among MRs. Internal medicine MRs and their preceptors were surveyed about their advice-seeking relationships and their self-perceptions regarding the adoption of the aforementioned leadership styles. For each actor in the network, leadership style data and network metrics were used to generate two scores, respectively representing LAI and LAD. The results gave rise to a four-quadrant model that allows to identify different manifestations of informal leadership, setting out a basis for targeted educational interventions.

## Theoretical approach

### Leadership theories considered in this study

As previously mentioned, four leadership theories are particularly relevant to medical residents: transformational, relational, adaptive, and resilient [35–38]. Transformational and relational leadership address the routine leader–follower dynamics of residency, emphasizing trust-building, goal alignment, and sustained motivation across rotations and shifts [35, 36]. In contrast, adaptive and resilient leaderships highlight the capacity of residents to guide teams through complexity, uncertainty, and acute disruptions in care [37, 38]. Table 1 presents a summary of these four theories.

**Table 1:** Main characteristics of leadership theories considered in this study

Leadership Theory	Transformational	Relational	Adaptive	Resilient
Unit of analysis	Leader–follower relationship	Team dynamics	Team dynamics	Leader–follower relationship
Leader's approach	Visionary and inspiring, creating an environment of innovation and commitment [41]	Encourages open communication, empathy, and participative decision-making [42]	Challenges followers, encouraging continuous learning and experimentation [43]	Focus on emotional intelligence and stress management to cope with crises [44]
Definition	Inspires followers to achieve significant changes through charisma, vision, and intellectual stimulation [35]	Emphasizes building strong, trust-based relationships with followers to enhance collaboration and mutual growth [36]	Mobilizes teams to face challenges and find innovative solutions through adaptation [43]	Sustains effectiveness in crisis by managing stress and fostering team cohesion [44]
Focus	Transforms followers and aligns individual and organizational goals [35]	Focuses on interpersonal connections, emotional intelligence, and shared goals [45]	Solves problems that require learning and cultural changes [43]	Focuses on interpersonal connections, emotional intelligence, and shared goals [45]
Objective	Drives long-term organizational and personal change [35]	Fosters a culture of inclusivity, trust, and teamwork to achieve sustainable outcomes [42]	Empowers teams to face and solve unknown challenges [43]	Restores and sustains organizational performance and cohesion during crises [45]

### Social network analysis metrics

SNA applies core concepts such as nodes (individuals), ties (relationships), and quantitative metrics to interpret social structures [46, 47]. For nodes, these metrics include degree, closeness, and betweenness centrality. Degree centrality reflects direct connections and popularity [48, 49]. Closeness centrality indicates accessibility within the network [48, 50]. Betweenness centrality highlights roles in mediating interactions and controlling information flow [48, 51]. At the network level, density measures overall connectivity among all nodes [48]. Specialized software, such as UCINET, calculates these metrics, while the NetDraw extension provides visual representations [52, 53]. These metrics and their variations can be interpreted in the context of informal leadership. For instance, in-degree centrality counts the number of ties directed towards a node, while in-closeness centrality measures the average shortest path from other nodes that seek support to that node. Both are reasonable proxies for informal leadership, as such individuals are often sought out by peers and are easily accessible [13]. In turn, out-closeness centrality, defined as the average shortest path length from a node to all others it supports, relates to the capacity to disseminate information across the network [54]. Fast and effective communication, reflected in high out-closeness centrality, is a characteristic of informal leadership in dynamic environments such as hospitals [1, 23]. The use of network metrics as proxies of other concepts is a common approach in SNA—e.g., Bertoni et al. [30] linking SNA metrics to organizational resilience. In fact, previous studies used SNA metrics as proxies for leadership theories. For instance, Bono and Anderson [55] show that transformational leadership relates to high in-degree.

### Associations between leadership styles and SNA metrics

Leadership styles are enacted through interaction patterns that can be reflected in positions within advice-seeking networks. For instance, relational leadership can be linked to brokerage and information mediation within the network [28, 36], which is logically associated with betweenness centrality. Transformational leadership is linked to being consistently sought out as a trusted source of guidance [35, 55], which is associated with in-degree centrality. Adaptive leadership is related to rapidly disseminating information under uncertainty [56, 57], which is associated with out-closeness centrality. Resilient leadership is linked to efficiently receiving information under pressure [30, 38], which is associated with in-closeness centrality. Table 2 summarizes these linkages between leadership styles and SNA metrics and the rationale for each pairing.

**Table 2:** Leadership styles and their proxies network metrics

Leadership style	Description	Proxy network metric
Relational	Their role in promoting collaboration reflects the core of relational leadership, which values social integration, cross-disciplinary interaction, and collective learning [28, 36] *Concise descriptor*: information broker	*Betweenness centrality*: quantifies how often a node lies on the shortest paths between other nodes, highlighting control over information flow [54, 58]
Transformational	These individuals are consistently sought out for their knowledge, vision, and ability to guide others. Their influence is built on trust, expertise, and a commitment to developing team members as mentors [35, 48, 55] *Concise descriptor*: trusted advisor	*In-degree centrality*: measures the number of incoming connections, directly indicating who is sought after [54, 58]
Adaptive	The ability to quickly disseminate information and tailor messages during times of uncertainty and crisis is central to adaptive leadership [29, 56, 57] *Concise descriptor*: rapid information disseminator	*Out-closeness centrality*: measures how quickly a node can reach all other nodes in the network [54, 58]
Resilient	The ability to quickly capture and process relevant information under pressure is a key trait of resilient leadership, enabling effective decision-making in high-stress situations [30, 38, 59] *Concise descriptor*: efficient information receiver	*In-closeness centrality*: measures how quickly a node can be reached by information originating from others [54, 58]

It is important to note that these linkages are approximations and thus the network metrics do not capture all dimensions of each leadership style. For example, resilient leadership involves effective decision-making under high stress situations. Although high in-closeness centrality suggests that decision-makers benefit from quickly receiving information, this falls short of indicating how effectively such information is used. Similar reasoning applies to the relationship between out-closeness centrality and adaptive leadership.

## Methods

### Overview of the hospital’s internal medicine service

The Internal Medicine Service (IMS) is situated within a large university hospital in Brazil. The hospital provides an average of 400,000 in-person consultations, 40,000 teleconsultations, 40,000 surgical procedures, 300 transplants, and 2,800 births annually. The institution employs 6,215 staff members, including 518 faculty, and supports 2,195 undergraduate students, 553 medical residents, and 108 multi-professional residents across all departments.

The IMS is staffed by 25 attending physicians (preceptors), seven of whom also hold university faculty positions. The residency program comprises 25 first-year residents (R1), 26 s-year residents (R2), and one third-year resident (R3), in addition to 21 rotating residents who complete one-month rotations at the IMS. Each month, 12–14 s-semester students and 15 final year interns participate in clinical activities within the service. The IM residency program has a mandatory duration of two years, with an optional third year available through a separate selection process.

In terms of infrastructure, the IMS has a reception area, separate rooms with computers for residents and preceptors, and meeting rooms, equipped with computers, televisions, and whiteboards, used for rounds. Residents also use equipment from other departments and hospital wards depending on their care activities. The work is organized into teams of up to eight members: one preceptor, one R2, two R1s, two interns, and one or two 5th-semester students. Residents work on internal medicine teams and rotate through different areas of the hospital (e.g., emergency room, intensive care unit, outpatient clinic, and wards), using the IMS space for computer access, meetings, and theoretical activities.

The internal medicine teams conduct daily rounds to review and discuss patient cases, both within the IMS facilities and at the point of care. The IMS also interacts with various specialties and multidisciplinary teams, including nursing, pharmacy, and nutrition. Seasonal variations affect patient flow and the types of illnesses encountered (e.g., increased respiratory cases during winter), requiring efficient resource management. The service manages a highly diverse patient population, often presenting multiple decompensated conditions. These contextual factors, such as high resident turnover and limited time spent in the IMS office, may influence interpersonal and professional interactions within the service.

### Data collection

The SNA questionnaire was applied to both preceptors and residents at the internal medicine service. Before receiving the questionnaire, participants were provided with the project title, average time for completing the form, the anonymity statement and a link containing more details on the project, ethics and project responsible contacts in case of doubts. They were then required to give informed consent by selecting *“Yes”* or *“No”* to the question *“Do you agree to participate in this research?”* The questionnaire was only available for those who marked *“Yes”* and blocked for those who marked *“No”*.

The survey comprised three sections with 15 questions in total: (i) demographic information about participants (6 questions), (ii) advice-seeking interactions (1 question), and (iii) participants’ perceptions on how they identify with the four leadership styles (8 questions). The 15 questions were administered to both preceptors and residents. Section (ii) (advice-seeking interactions) used a standard roster-based advice-seeking question, which is widely used in SNA research in healthcare [60] and also had been used in other SNA studies with other clinicians at the same hospitals [30, 61]. Because SNA analysis necessarily requires identification of respondents [30, 60], the survey was confidential but not anonymous. Respondent names were used only for initial data storage, and then the dataset was de-identified prior to analysis and reporting of results. Section (iii) (participants self-perceptions on leadership styles) was developed by a theory-driven mapping, in which the eight self-perception statements (two per leadership style) were derived from the core characteristics summarized in Table 1. Leadership style labels (i.e., transformational, relational, adaptive, and resilient) were not shown to respondents to reduce bias toward a particular style. The complete survey is in Supplementary File 1.

The survey was pilot tested with two preceptors prior to launch to assess clarity, survey flow, and completion time. The pilot resulted in minor wording refinements. Nevertheless, the two pilot responses were excluded from the final sample and those two preceptors filled out the survey again when it was administered to the entire population. Their new responses were nearly identical to those obtained during the pilot, reinforcing the reliability of the questionnaire. Two main groups were targeted: preceptors (P), and residents, from first, second, and third year (R1, R2, R3). Residents from other specialties rotating through the IMS were excluded due to high turnover. After human subjects’ approval was obtained from the ethics committee, the survey invitation was sent to all 77 physicians via Qualtrics, a platform commonly used in SNA, and participants were contacted primarily through institutional e-mail. The survey yielded 68 responses – 46 residents and 22 preceptors (overall response rate of 88.3%), exceeding the rate of 75% recommended by Borgatti, Carley, and Krackhardt [58] for ensuring valid and reliable SNA metrics results. Also, Brass and Borgatti [62] point out that a response rate of 80% suffices to capture the most important network characteristics. All 68 respondents provided informed consent by selecting “*Yes*” to the consent question, and no respondent selected “*No*”. Non-participation occurred through non-response as 9/77 invited physicians did not submit the questionnaire. Reasons for non-response were not systematically collected. However, some non-responders informally provided justifications – e.g., one preceptor was on vacation, another was ill during the survey period, and two residents reported time constraints related to jobs extra to residency duties.

Another questionnaire, presented in Supplementary File 2, was developed to capture perceptions of the relative importance of the four leadership styles in training future IMS leaders (1 question). This information was used to determine weights for leadership styles in the LAI and LAD computations. This questionnaire was administered exclusively to the 22 preceptors who submitted their responses to the SNA survey, and it was applied separately from this survey, in a later moment. Nineteen valid responses were received, corresponding to an 86.4% response rate. The non-responses can be attributed to questionnaire fatigue and time constraints among busy clinicians.

### Data analysis

LAD was operationalized using the advice-seeking network data derived from question 7, and responses to the second questionnaire on the relative importance of leadership styles. SNA metrics were generated by UCINET software, and the network graphic (i.e., sociogram) was obtained using the NetDraw extension. Responses to question 7 were used to build an adjacency matrix in which each cell ${x}_{jk}$ represented how frequently participant $j$ sought advice from person $k$. Then, for each individual, four centrality metrics that captured complementary dimensions of network influence were computed: in-degree, betweenness, out-closeness, and in-closeness. As presented in Sect. 2.3, these four metrics are proxies of the four leadership styles as follows: in-degree for transformational leadership, betweenness for relational leadership, out-closeness for adaptive leadership, and in-closeness for resilient leadership.

All centrality metrics were normalized to allow comparison and to enable their combination into a novel composite score denoted as the Leadership Score (${LS}_{k}$). ${LS}_{k}$ is defined as follows: let ${w}_{i}$ denote the importance weight assigned to leadership style $i$, computed as the sum of the reciprocals of rank positions attributed to each style $i (i=1,\dots ,4)$ by the 19 preceptors who responded section (iv) of the survey. Let ${n}_{ik}$ denote the network metric of individual $k$ associated with leadership style $i$ (see Table 2). The Leadership Score is then given by:

1 $${LS}_{k}={\sum }_{i=1}^{4}\left({W}_{i}\times {n}_{ik}\right),k=1,\cdots ,68$$

The weights were normalized such that ${\sum }_{i=1}^{4}{w}_{i}=1$. Equation (1) thus represents a weighted aggregation of selected network metrics derived from the advice-seeking relationships [section (ii) of the survey], scaled by the relative importance of each leadership style elicited in section (iv). The normalization process of weights was conducted as follows. Initially, for each leadership style, preceptors ranked its importance from 1 (most important) to 4 (least important). Then, this rank was translated into numerical values: a rank of 1 provided by preceptor *i* received a value of 1.00; a rank of 2 received 0.50; a rank of 3 received 0.33; and a rank of 4 received 0.25. The values assigned by all preceptors were then summed for each leadership style, resulting in one aggregated score per style. Finally, these aggregated scores were converted into relative weights by dividing each style’s score by the total sum across the four styles. For example, the sum of values for relational leadership was 12.07, and the total sum across all four styles was 42.11. Thus, the weight assigned to relational leadership was 12.07/42.11 = 0.29, representing its relative importance in the Internal Medicine context. This weighting procedure reflects the context-dependent demands of complex hospital settings, in which different clinical situations require different leadership styles.

LAI was operationalized using questions 8 to 15 representing participants’ self-assessed development across the four leadership styles, and results from the second questionnaire on the relative importance of leadership styles. Questions 8 to 15 comprised eight statements capturing the core aspects of the four leadership styles – two statements per style, with agreement rated on a 5-point Likert scale. For each participant, an LAI score was calculated by summing the means of each leadership style—comprising four different means per individual—and multiplying them by the corresponding weights assigned by the preceptors. The LAI score was the sum of these four weighted means per individual.

After calculating the LAI and LAD scores for each participant, all individuals were plotted in a two-dimensional graph with LAI scores on the horizontal axis and LAD scores on the vertical axis. To distinguish between “low” and “high” score values, the sample means were used as cut-off points, thereby reflecting the characteristics of the observed system. As such, it was possible to delineate four quadrants, named as follows: high LAI and high LAD (established leaders); low LAI and high LAD (humble leaders); high LAI and low LAD (aspirational leaders); and low LAI and low LAD (latent leaders). Residents positioned in the established and humble quadrants were regarded as informal leaders. These quadrant labels convey formative and developmental profiles, rather than static and permanent classifications, as they reflect a snapshot of the moment when data was collected.

In the last step, each LAI-LAD quadrant was linked to a set of targeted educational interventions. These intervention proposals were based on logical reasoning related to each quadrant characteristics (e.g., participants with low LAI and low LAD scores should require more extensive training) as well as on insights from the literature.

The aforementioned data analysis procedures were applied for the modelling of two boundary conditions: (i) the full network comprised of residents and preceptors; and (ii) a residents-only network. The full network provides the more accurate modelling for the identification of informal leaders, due to two main reasons: (i) the presence of preceptors reflects the real clinical system, and consequently it is crucial for modelling LAD, where residents seek guidance from preceptors not necessarily owing to hierarchical obligations; and (ii) informal leadership of residents depends on how they interact with preceptors—e.g., some residents serve as bridges between other residents and preceptors, and residents strongly connected to preceptors can gain more opportunities to develop their leadership skills. In addition, the inclusion of preceptors provides a benchmark that allows analyzing whether some residents reach levels of leadership comparable to preceptors.

The residents-only network was useful to assess the stability of residents’ LAD scores and quadrant assignment across network boundaries. Note that because quadrant cut-offs were defined as sample means, the cut-off values differ when computed for the full network and for the residents-only network.

## Results

### Demographic characteristics of participants

Table 3 presents the main characteristics of the 68 participants. Forty-two of them were male (61.8%) and 26 females (38.2%). They were evenly split between preceptors (22; 32.4%) and first-year residents (22; 32.4%), with a similar proportion of second-year residents (23; 33.8%), and a single third-year resident (1; 1.5%). Regarding clinical experience, 11 participants (16.2%) had less than one year as a doctor, 21 (30.9%) had 1–2 years, and 19 (27.9%) had more than 10 years, indicating a mix of junior and senior physicians. Experience in internal medicine was shorter, with 29 participants (42.6%) reporting less than one year and 21 (30.9%) between 1–2 years, while only 9 (13.2%) had more than 10 years in the specialty. Experience is reported in this study as a descriptive characteristic to contextualize group profiles. As demonstrated by the results detailed next, it is not a determinant of informal leadership.

**Table 3:** Demographic characteristics of study participants

**Gender**	***n* (%)**
Female	26 (38.2%)
Male	42 (61.8%)
Staff	*n* (%)
Preceptors (P)	22 (32.4%)
First-year residents (R1)	22 (32.4%)
Second-year residents (R2)	23 (33.8%)
Third-year residents (R3)	1 (1.5%)
**Years of experience as a doctor**	***n* (%)**
Less than 1	11 (16.2%)
1–2	21 (30.9%)
2–3	7 (10.3%)
3 −5	5 (7.4%)
6–10	5 (7.4%)
More than 10	19 (27.9%)
**Years of experience in Internal Medicine**	*n* (%)
Less than 1	29 (42.6%)
1–2	21 (30.9%)
2–3	1 (1.5%)
3 −5	3 (4.4%)
6–10	5 (7.4%)
More than 10	9 (13.2%)
Total	68 (100.0%)

### The relative importance of leadership styles

Before computing LAI and LAD scores, the relative importance of the four leadership styles from preceptors’ rankings were estimated according to the procedures described in Sect. 3.3. As a result, the following normalized importance weights (w) were obtained to each leadership style: relational (w = 0.29), transformational (w = 0.28), adaptive (w = 0.23), and resilient (w = 0.20). Although the four styles were clearly important, relational and transformational styles ranked slightly higher than adaptive and resilient styles. These results can be due to the multidisciplinary and technically complex nature of internal medicine, which requires teamwork and technical expertise, attributes respectively valued by relational and transformational styles.

### Leadership-as-imagined (LAI)

Scores for LAI related to the self-perception questions ranged from 3.07 to 5.00, with an average of 4.20 for the entire sample. Seventeen out of the 46 residents, representing 25% of the entire sample, presented LAI values higher than the overall mean, indicating that they perceived themselves as displaying significant leadership skills. Table 4 shows the ten highest LAI scores, which included six preceptors, one first-year resident and three second-year residents – the full list of LAI scores for the 68 participants is presented in Supplementary File 3. These residents feel highly confident about their ability to mobilize colleagues, communicate clearly, and manage difficult situations, even at relatively early stages of residency.

**Table 4:** The 10 highest self-perception averages (LAI)

ID	LAI (S.D.)
P1	5.00 (0.21)
P18	5.00 (0.21)
P22	5.00 (0.21)
R1.15	4.89 (0.24)
P17	4.80 (0.30)
R2.5	4.80 (0.30)
P5	4.79 (0.27)
P10	4.79 (0.27)
R2.20	4.79 (0.27)
R2.23	4.79 (0.27)

*LAI* Leadership-as-imagined, *S.D.* Standard Deviation

On the other hand, 27 out of 46 residents (58.7% of the residents) presented LAI scores lower than the overall mean, indicating that they did not see themselves as leaders. However, that does not necessarily imply an absence of leadership potential but can rather suggest an acute awareness of their position as learners or followers.

### Leadership-as-done (LAD)

LAD was assessed using the composite Leadership Score (${LS}_{k}$) derived from the advice-seeking network and the importance weights provided by preceptors. The LAD results presented in this section were computed for the full advice-seeking network including both residents and preceptors. ${LS}_{k}$ values ranged from 1.45 to 3.97, with an average of 2.22, which was also used as the cut-off between low and high LAD. Table 5 presents the ten highest LAD scores, including their respective network metrics rescaled to a 5-point Likert scale – the full list of LAD scores is presented in Supplementary File 3. In this group, there were five preceptors, two first-year residents, and three second-year residents. There was only one actor (R2.5) who appeared as a top 10 scorer for both LAI and LAD, suggesting a significant gap between these two dimensions. This point can also be further illustrated by R2.23 who obtained the 10th highest LAI score and ranked 34th for LAD, with a LS = 2.06. R2.5 and R2.23 exemplify that two same year residents may have substantially different leadership profiles (see characterization of leadership groups, in Sect. 4.5). Although the identification of the reasons for these differences is beyond the scope of this study, some possible explanations can be related to personality traits, mentorship by preceptors, and educational background prior to medical school.

**Table 5:** The 10 highest Leadership Scores (LAD)

ID	Network attributes				Non-network attributes
	In-Degree	Betweenness	Out-Closeness	In- Closeness	LS (S.D.)
P9	3.31	5.00	4.26	3.06	3.97 (0.35)
P12	4.02	4.05	4.56	2.59	3.87 (0.30)
R2.14	2.42	4.86	3.27	5.00	3.84 (0.33)
P4	4.56	3.34	4.78	2.28	3.80 (0.35)
P21	4.73	2.18	4.83	1.94	3.46 (0.43)
P7	5.00	1.46	5.00	1.65	3.31 (0.53)
R2.5	2.07	3.50	3.63	3.63	3.16 (0.18)
R2.3	2.78	2.21	3.50	2.99	2.82 (0.10)
R1.6	1.62	2.30	3.06	4.54	2.74 (0.19)
R1.8	1.98	2.50	3.50	3.13	2.71 (0.11)

*LS* Leadership Score, *S.D.* Standard Deviation

Preceptor P9 had the highest LAD score (LS = 3.97), followed closely by P12 (3.87), R2.14 (3.84), P4 (3.80), and P21 (3.46). These actors combine high in-degree (for example, P7 with 46 incoming advice nominations, P4 with 41, and P21 with 43) with high betweenness and closeness values, acting as bridges between different parts of the service and as accessible sources of information for colleagues. By contrast, individuals presenting the lower LAD scores had few incoming ties (for example, R1.15 with no incoming ties, R1.2 with one, and R2.2 with four), remaining at the edges of the network. Although these participants contribute to patient care and team functioning, they are not widely recognized as advice sources.

**Fig. 1:**
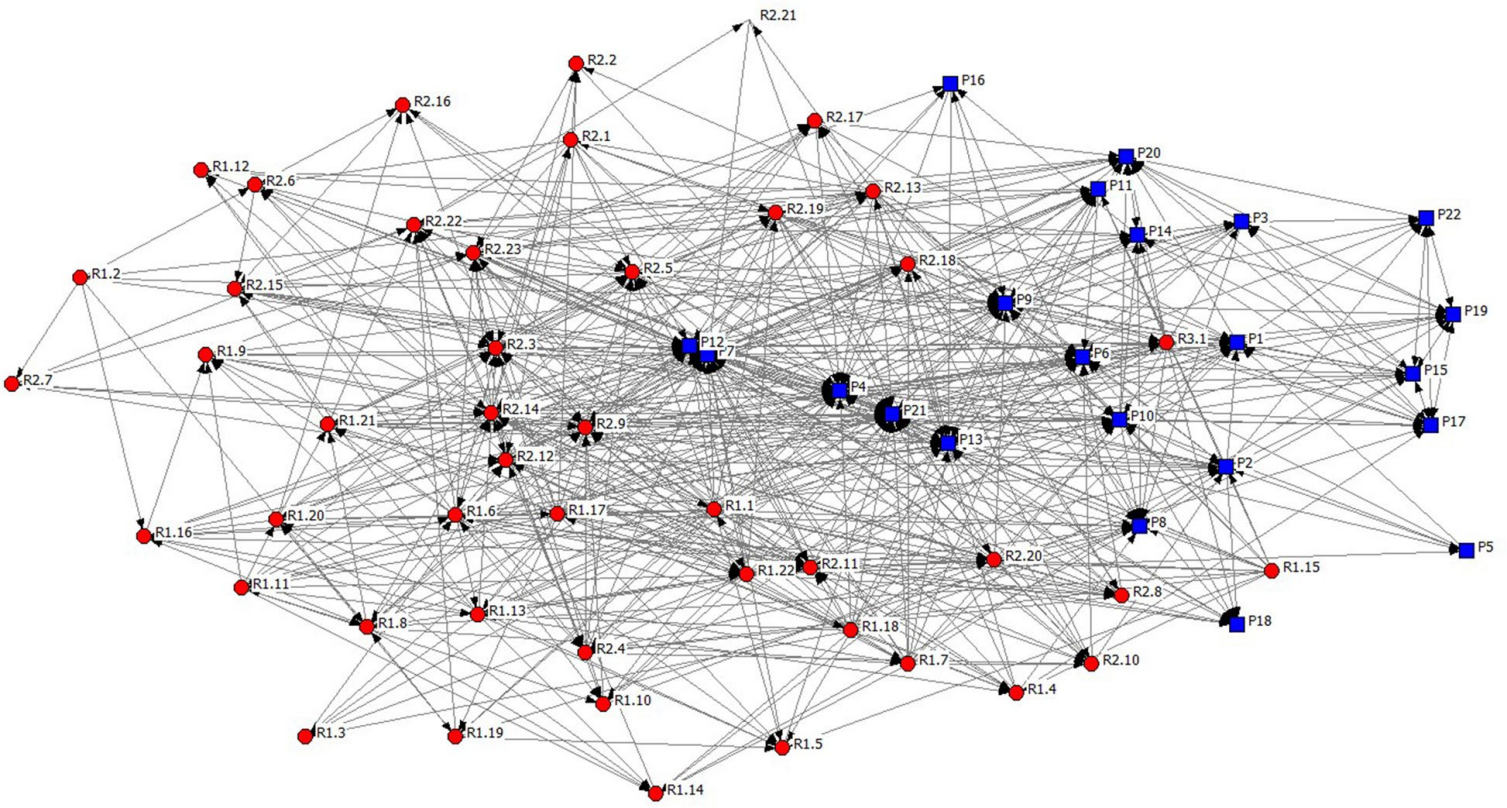
Sociogram of the network. Note: blue squares represent preceptors and red circles represent residents

Responses to the advice-seeking question also informed the development of the sociogram in Fig. 1, which shows a highly connected network. Although advice flows in multiple directions and across hierarchical levels, the sociogram shows a clear centralization around a subset of highly connected actors, while others remain in more peripheral positions. Such positions are occupied by both residents and preceptors as indicated in Fig. 1.

### Characterization of leadership groups

**Fig. 2:**
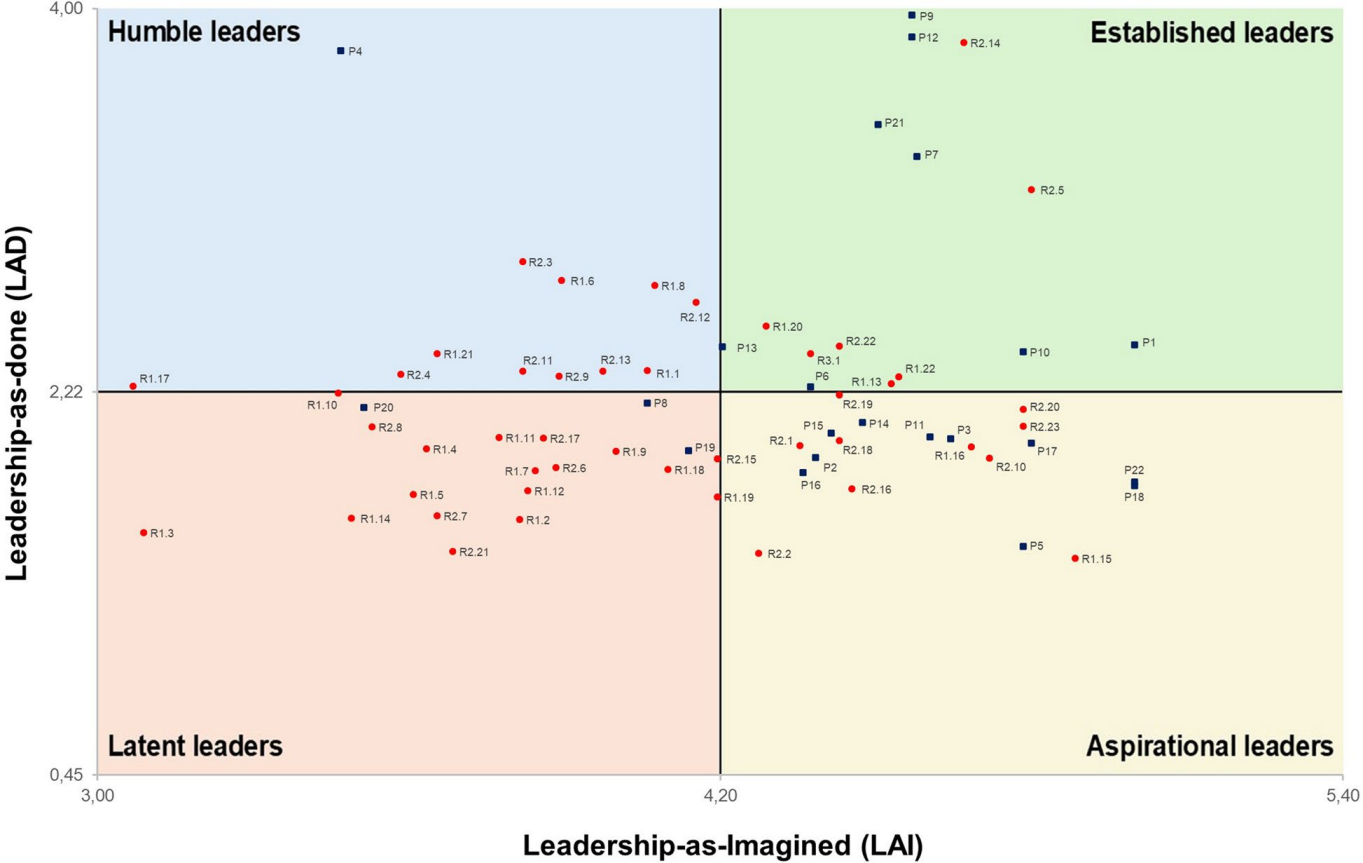
Positioning of participants according to their LAI and LAD scores

Figure 2 plots all individuals according to their LAI and LAD scores for the entire network. Quadrants are partitioned based on the average values of LAI and LAD, and their rationale is described in Table 6.

**Table 6:** The four quadrants of leaders

Quadrant	LAI	LAD	Definition
Established leaders (high LAI, high LAD)	≥ 4.20	≥ 2.22	These individuals strongly perceive themselves as leaders and occupy central positions in the advice-seeking network
Humble leaders (low LAI, high LAD)	< 4.20	≥ 2.22	These individuals are central in the advice-seeking network, but do not regard themselves as skilled leaders
Aspirational leaders (high LAI, low LAD)	≥ 4.20	< 2.22	These individuals strongly perceive themselves as leaders yet occupy relatively peripheral positions in the network. They express leadership aspirations, which have not yet been fully exploited by their peers
Latent leaders (low LAI, low LAD)	< 4.20	< 2.22	These individuals neither tend to see themselves as leaders nor occupy central positions in the advice-seeking network

The cut-off points for the quadrants corresponded to the average of LAI and LAD scores

Table 7 presents the distribution of participants according to the quadrants. Among the 46 residents, 7 (15.2%) were classified as established leaders, 11 (23.9%) as humble leaders, 10 (21.7%) as aspirational leaders, and 18 (39.1%) as latent leaders. These mixed profiles further reinforce the argument of this paper regarding the need for targeted and to some extent customized, educational interventions.

**Table 7:** Distribution of participants according to the four quadrants

Quadrant [Quantity (%)]	Preceptors [Quantity (%)]	Residents [Quantity (%)]		
		*R1*	*R2*	*R3*
Established leaders 15 (22.0%)	8 (53.3%)	3 (20.0%)	3 (20.0%)	1 (6.7%)
Humble leaders 12 (17.6%)	1 (8.3%)	5 (41.7%)	6 (50.0%)	0 (0.0%)
Aspirational leaders 20 (29.4%)	10 (50.0%)	2 (10.0%)	8 (40.0%)	0 (0.0%)
Latent leaders 21 (31.0%)	3 (14.3%)	12 (57.1%)	6 (28.6%)	0 (0.0%)

Regarding the professional role, established leaders included 8 preceptors and 7 residents (3 R1s, 3 R2s, and 1 R3). These residents stand out as the main informal leaders, and their average experience time in internal medicine residency was 1.5 years. Humble leaders included 1 preceptor and 11 residents (5 R1s and 6 R2s); residents’ average experience time in internal medicine residence was 1.1 years. While humble residents are also informal leaders, their lower experience and lower LAI scores allow to hypothesize that their leadership effectiveness is lower in comparison to the established leaders. Aspirational leaders included 10 preceptors and 10 residents (2 R1s and 8 R2s). Possible hypotheses for the high incidence of preceptors in this group are that their leadership effectiveness is low and that they are experts in highly specialized fields, rather than being generalists with a wider potential for the provision of advice. Among residents, most aspirational leaders were R2s, suggesting that some junior physicians feel ready to lead but are still building their network influence within the service. Latent leaders comprised 3 preceptors and 18 residents (12 R1s and 6 R2s), with two-thirds of them in the first year. This may reflect limited involvement in leadership roles during their current stage of clinical training. It is noteworthy that there were two preceptors in this group, suggesting that their formal responsibility for supervision is not translating into influence within the advice-seeking network.

### Comparison between full network and residents-only network

To assess how network boundary definition affects residents’ LAD values and LAI-LAD quadrant assignment, we compared the full-network (preceptor-resident; *n* = 68) with a residents-only network (resident–resident; *n* = 46). The residents-only adjacency matrix retained the resident–resident ties and excluded ties involving preceptors (56.1% of all ties in the full network). In the full network, preceptors accounted for 6 of the 10 highest LAD values (see Table 5), so excluding preceptors removes several high-centrality advice ties and substantially oversimplifies the actual network complexity.

Thirty-five of the 46 residents (76.1%) remained in the same quadrant, whereas 11/46 (23.9%) moved to an adjacent quadrant after recalculating LAD metrics and quadrant cut-offs under the residents-only boundary. Specifically, the observed transitions were: aspirational to established (3 residents), humble to established (2 residents), humble to latent (2 residents), latent to humble (2 residents), latent to aspirational (1 resident), and established to aspirational (1 resident). A possible hypothesis for these transitions is that, under conditions of complete and extended absence of preceptors, a minority of residents will be most affected through diverse mechanisms. For example, some of them can develop self-awareness and self-confidence in their skills in the absence of preceptors (e.g., those transitioning from humble to established); others may lose status as bridges between residents and preceptors, being repositioned as aspirational rather than established leaders; others can simply be forced to lead due to the absence of preceptors, transitioning from latent to humble leaders; and others can receive more opportunities to lead their peers and apply their skills, moving from aspirational to established. The complete side-by-side comparison (full network versus residents-only, LAI, LAD, and quadrant classification) is provided in Supplementary File 4.

## Discussion

### On the alignments and misalignments between LAI and LAD

Alignment between LAI and LAD occurs for established leaders and latent leaders. Considering the full network with residents and preceptors, there were 8 preceptors and 7 residents in the established leaders’ quadrant. Even though residents and preceptors may be established leaders, they might experience this role very differently in terms of workload, emotional strain, and risk of burnout [12, 63]. Furthermore, the notion of established leaders resembles the concept of key players in social network analysis. According to Borgatti et al. [58], key players are optimally positioned to quickly diffuse information, attitudes, behaviors or goods and/or to quickly receive the same. As a drawback, an overreliance on a few key players can make the network performance vulnerable when they are unavailable [1].

Alignment between LAI and LAD also occurred for latent leaders. This quadrant contained a large proportion of first-year residents (26.1% of the entire group of residents), which is consistent with the steep learning curve in early residency and with studies showing that the new resident often feels unprepared to lead teams or manage crises [7, 64]. From an educational perspective, this group should not be labelled as “weak”, but rather as individuals whose leadership identity and network role are still emerging and developing. Recognizing latent leaders as being at an early stage of residents’ identity construction reframes them from “followers” to prospective leaders in formation [9, 21].

Misalignment between LAI and LAD appears among aspirational leaders, who see themselves as leaders yet are not frequently sought by peers for advice. This group included 10 of 22 preceptors (45.4% of the entire preceptors’ group) and 10 of 46 residents (21.7%), of whom 8 were in their second year of residency. LAI not translated into LAD can be due to structural barriers related to rotation patterns, shift schedules, task assignment, or hierarchies [65, 66]. The predominance of preceptors and R2s in this quadrant suggests that experience, by itself, is not enough to ensure network legitimization. Moreover, aspirational leaders reflect findings of past studies indicating that physicians tend to overestimate their non-technical performance [13, 67, 68].

Misalignment between LAI and LAD also occurs among humble leaders. They comprised 4.5% of all preceptors (1/22) and 23.9% of the residents (11/46), indicating that they are central points of reference in the advice-seeking network despite not perceiving themselves as skilled leaders. Studies of leadership identity point out that influence can be attributed to individuals before they feel fully competent in that role [14]. This mismatch can be a source of stress and frustration for these individuals.

The alignments and misalignments between LAI and LAD can also be interpreted as an identity-building process through which residents recognize themselves as leaders – and are also recognized by their peers. In this view, LAI reflects leadership identity claims, whereas LAD reflects leadership identity grants expressed through recurring advice-seeking ties. This insight is consistent with the idea that leadership identity is socially constructed and negotiated through interaction rather than solely possessed as an individual attribute [13, 33]. Established leaders reflect established identity, while latent leaders are at an early stage of leadership identity development [13, 32]. The aspirational leaders’ quadrant exemplifies an identity-recognition gap. In this group, individuals claim leadership identity but do not receive network-based grants, which can be shaped by contextual conditions such as rotations, shift structures, task allocation, and hierarchical coordination demands [27, 29, 30]. For humble leaders, leadership identity is socially granted before it is fully internalized, potentially increasing stress when residents become central advice targets without self-confidence or perceived competence [13, 16]. These patterns suggest that leadership development in residency can support identity formation.

### Targeted interventions by leadership groups

The four-quadrant framework allows to consider suitable directions for leadership development for each group. This idea aligns with the need for context-sensitive and data-driven approaches to leadership development in health education [15, 21, 69]. Leadership initiatives tend to be more effective when they are aligned with participants’ baseline experience, identity stage, and contextual constraints [14, 21, 70]. The quadrants provide a diagnostic starting point to discuss how existing educational strategies such as mentoring, feedback, and team training could be prioritized or tailored for established leaders, humble leaders, aspirational leaders, and latent leaders.

Across the health professions education literature, leadership skills training encompasses educational approaches designed to develop observable leadership behaviors in clinical work (e.g., coordination, feedback, negotiation, and team-based decision-making) through guided practice and feedback [11, 69]. Such approaches typically combine complementary components, including: (i) teaching (e.g., short courses or workshops); (ii) mentoring or coaching of leadership roles in hospital settings; (iii) team-based routines and tools that foster coordination and shared decision-making; and (iv) structured feedback and reflection to calibrate self-perceptions and reinforce effective leadership behaviors [11, 52, 69]. Improvements in leadership skills are typically assessed through changes in observable behavior, team-based feedback (e.g., supervisor and peers), and performance in workplace, rather than relying only on self-reports [11, 69].

For established leaders, interventions should focus on advanced training. Established leaders are natural candidates to play central roles in educational and quality-improvement activities, given that they are both self-identified and network-recognized as key sources of advice. Engaging such residents in roles that combine formal learning with practical responsibilities – for example, co-facilitating debriefings, mentoring junior residents, or contributing to curricular refinements – is associated with gains in leadership competences and organizational outcomes [21, 70, 71]. These leadership development opportunities have been associated with gains in leadership capability and improved organizational outcomes [17, 52, 69]. At the same time, established leaders may become overwhelmed by these responsibilities, requiring initiatives that support personal resilience, wellbeing, and sustainable engagement in leadership roles [63, 72].

For the latent leaders’ group, interventions may focus on providing supervised leadership experiences as they are at early stages of leadership identity construction. Indeed, this group can benefit most from progressive participation in everyday clinical leadership tasks, explicit role-modelling, and coached opportunities to lead small, bounded activities (e.g., case presentations, coordination during rounds, or structured handover or plan alignment), with structured feedback to build leadership competence and self-confidence [17, 32, 69]. The healthcare literature highlights the importance of this approach along with psychologically safe environments for speaking up such as by creating reflexive spaces [6, 7, 73]. These mechanisms support leadership maturing over time.

Interventions for aspirational leaders may provide opportunities for increasing the network centrality of these individuals. From an educational perspective, misalignment in this quadrant should not be interpreted necessarily as “overconfidence”, but rather as a sign that these individuals are actively engaging in leadership identity construction without being recognized by their team as informal leaders [14, 74]. For this group, structured mentoring relationships should be combined with opportunities for taking leadership roles such as co-leading ward rounds, leading case discussions, or taking responsibility for small quality-improvement projects and team meetings. These experiences can increase their chances of being legitimized by the network, progressing toward the established-leaders quadrant [8, 21, 75].

Interventions for humble leaders may focus on enhancing leadership skills and confidence so that their self-perceptions more closely match the high demand for advice they receive within the network. This group was composed mainly of first- and second-year residents, which raises concerns about the concentration of coordination demands in some inexperienced individuals who are not confident in their skills. For this group, the literature points to the importance of combining strategies to promote self-confidence, reduce distress, recognition and feedback that makes it clear their central network role and how to cope with it [71, 75, 76]. Hence, interventions can prioritize (i) mentoring and psychological support to reduce role-related stress associated with being relied upon early, and (ii) guided practice in coordination and communication behaviors to strengthen perceived competence [52, 56, 68].

Additionally, there is an implication that applies to all quadrants and connects to the way LAI and LAD were combined in this study. Sharing aggregated and (where appropriate) individualized results of LAI and LAD with residents through feedback sessions or facilitated debriefings may help participants to reflect on how they see themselves as leaders, and how they are seen by others in the advice network. This type of feedback is consistent with indications that structured reflection can calibrate physicians’ self-perceptions and stimulate more realistic assessments of their non-technical competences [31, 66, 67]. By making the patterns of alignment and misalignment visible, network-based feedback may serve as a low-cost intervention that supports leadership identity development in all quadrants.

Finally, although the educational implications of this study are framed around residents, the LAI–LAD framework can guide targeted development for preceptors as well. In particular, preceptors in misaligned quadrants may also benefit from support mechanisms such as structured feedback on observable behaviors, coaching or mentoring, and psychologically safe reflective spaces to improve calibration between self-recognition and network recognition [11, 68].

## Conclusions

This research study addressed the knowledge gap regarding how formal leadership theories can inform the identification of informal leaders in medical residency, with the aim of supporting educational interventions. A novel approach based on a social network analysis survey is proposed, combining a traditional advice-seeking relationship question with tailored items on the adoption and importance of four leadership styles. Transformational, relational, adaptive, and resilient leadership concepts were translated into survey questions that were used to construct the LAI scores and to weigh the network centrality metrics that compose the LAD score.

By comparing LAI and LAD scores in the internal medicine study, this work demonstrates how expectations about leadership and actual influence patterns may either converge or diverge in a medical residency program. The LAI-LAD framework provides both a new descriptive taxonomy of leadership configurations and a novel diagnostic tool to identify informal leaders and where educational interventions are most needed.

This study has some limitations. First, the survey instrument was tested only in the internal medicine context. However, its structure and underlying logic are not context-specific and could be applied to other medical residency programs. A key adaptation for other contexts would be the selection of relevant leadership styles to be assessed. Second, this was a cross-sectional study and thus did not capture how networks and associated leadership profiles change over time – e.g., changes in training level. Future longitudinal research could monitor how residents’ LAI and LAD scores evolve throughout the entire residency, enabling interventions at different stages. Third, social network analysis alone provides limited insight into why a network assumes a particular configuration. Further research could incorporate qualitative data (e.g., from interviews) to explore underlying factors such as personal attributes of leaders and followers, leadership influence processes, and organizational context. Fourth, advice-seeking nominations can be affected by formal supervisory arrangements, as residents can preferentially consult assigned preceptors, potentially introducing hierarchical bias in the reported ties. To contextualize potential hierarchical effects in advice ties, the main results derived from the full network (residents and preceptors) were complemented by a residents-only comparison. Fifth, the LAD represented by the Leadership Score relies on the use of selected network metrics as proxies of leadership styles, not covering all elements of each style. Although the LAI survey questions were theory-derived and pilot-tested for clarity, formal psychometric evaluation (e.g., dimensionality, reliability, and convergent and discriminant validity) was beyond the scope of this study and should be addressed in future research. Sixth, because the survey was confidential but not anonymous, social desirability bias cannot be fully ruled out.

## Supplementary Information

Supplementary Material 1.

Supplementary Material 2.

Supplementary Material 3.

Supplementary Material 4.

## Data Availability

The data that support the findings of this study are available from the corresponding author, but restrictions apply to the availability of these data, which were used under license for the current study, and so are not publicly available. Data are, however, available from the corresponding author upon reasonable request and with permission of the Ethics Committee of Hospital de Clinicas de Porto Alegre.

## Abbreviations

IM: Internal Medicine
IMS: Internal Medicine Service
LAI: Leadership-as-imagined
LAD: Leadership-as-done
LS: Leadership Score
MRs: Medical residents
P: Preceptors (applied to all preceptors – e.g. P1, P2, P3)
R1: First-year residents
R2: Second-year residents
R3: Third-year resident
S.D.: Standard Deviation
SNA: Social Network Analysis
UCINET: UCINET software (used to calculate SNA metrics)
WAI: Work-as-imagined
WAD: Work-as-done
