# Supplementary material for: Identifying informal leaders among medical residents as a basis for educational interventions
Source: BMC Med Educ. 2026 Feb 28;26:560. doi: 10.1186/s12909-026-08918-0 (PMC13059413; doi:10.1186/s12909-026-08918-0)
Supplement: Supplementary file 1 — Supplementary Material 1. [file 12909_2026_8918_MOESM1_ESM.docx]

**Supplementary file 1:** SNA and leadership survey

| **Section name** | **Question #** | **Question name** | **Possible Responses** |
| --- | --- | --- | --- |
| Section I: For Preceptors and Residents | | | |
| Survey start | - | Survey questionnaire opens with the presentation of the project and Ethics Committee, providing a link to the Consent Term by the Ethics Committee of Hospital de Clínicas de Porto Alegre and a questions of agreement for participating in the study. | Yes / No |
| Demographic data | 1–4 | Name; Age; Sex; Role in the Internal Medicine department | Full name; Numeric value; Male / Female; Preceptor, Resident (R1–R3). |
|  | 5 | Years of experience in the medical field (general), excluding time as a medical student | Number of years |
|  | 6 | Years of experience in the Internal Medicine Service | Number of years |
| Section II: For Preceptors and Residents | | | |
| Name roster | 7 | From the list of names below, check those with whom you interact to obtain advice, information, or make work-related decisions | List of names of preceptors and medical residents from the IMS |
| Section III: For Preceptors and Residents | | | |
| Section entry text: In questions 8 to 15, indicate your level of agreement with the statements presented: | | | |
| Transformational Leadership | 8 | “I seek to inspire my team members, motivating them to develop their potential and exceed expectations, contributing beyond their usual responsibilities.” | Scores on a 5-point Likert scale: 1 - Strongly disagree; 2 - Partly disagree; 3 - Neutral; 4 - Partly agree; 5 - Strongly agree. |
|  | 9 | “I seek to promote a culture of change in my team, encouraging them to openly share their perspectives.” | Scores on a 5-point Likert scale: 1 - Strongly disagree; 2 - Partly disagree; 3 - Neutral; 4 - Partly agree; 5 - Strongly agree. |
| Relational Leadership | 10 | “I seek to develop and foster positive relationships among my team members, encouraging interaction and communication.” | Scores on a 5-point Likert scale: 1 - Strongly disagree; 2 - Partly disagree; 3 - Neutral; 4 - Partly agree; 5 - Strongly agree. |
|  | 11 | “I seek to encourage collaboration among my team members to achieve our common objectives.” | Scores on a 5-point Likert scale: 1 - Strongly disagree; 2 - Partly disagree; 3 - Neutral; 4 - Partly agree; 5 - Strongly agree. |
| Adaptive Leadership | 12 | “I encourage my team members to step out of their comfort zone and explore new solutions to the challenges we face.” | Scores on a 5-point Likert scale: 1 - Strongly disagree; 2 - Partly disagree; 3 - Neutral; 4 - Partly agree; 5 - Strongly agree. |
|  | 13 | “I can easily understand the complexity of situations, changing tactics or strategies as we adapt to new scenarios.” | Scores on a 5-point Likert scale: 1 - Strongly disagree; 2 - Partly disagree; 3 - Neutral; 4 - Partly agree; 5 - Strongly agree. |
| Resilient Leadership | 14 | “When an unexpected event occurs in my daily routine, I seek to prioritize managing my team’s stress level, maintaining emotional and mental balance.” | Scores on a 5-point Likert scale: 1 - Strongly disagree; 2 - Partly disagree; 3 - Neutral; 4 - Partly agree; 5 - Strongly agree. |
|  | 15 | “I can understand the variabilities that occur in my daily routine by monitoring, anticipating, and adjusting actions or resources according to imposed constraints.” | Scores on a 5-point Likert scale: 1 - Strongly disagree; 2 - Partly disagree; 3 - Neutral; 4 - Partly agree; 5 - Strongly agree. |
| End of survey | - | Closing remarks | - |
