# Supplementary material for: Identifying informal leaders among medical residents as a basis for educational interventions
Source: BMC Med Educ. 2026 Feb 28;26:560. doi: 10.1186/s12909-026-08918-0 (PMC13059413; doi:10.1186/s12909-026-08918-0)
Supplement: Supplementary file 2 — Supplementary Material 2. [file 12909_2026_8918_MOESM2_ESM.docx]

**Supplementary file 2:** Questionnaire for weighting the importance of leadership styles

| **Section name** | **Question #** | **Question name** | **Possible Responses** |
| --- | --- | --- | --- |
| Section I: For Preceptors only | | | |
| Survey start | - | Survey questionnaire opens with the presentation of the project and Ethics Committee, providing a link to the Consent Term by the Ethics Committee of Hospital de Clínicas de Porto Alegre and a questions of agreement for participating in the study. | Yes / No |
| Section entry text: Rank the four leadership styles presented from 1 to 4 in order of importance, where 1 indicates the most important style and 4 indicates the least important style. If you consider two or more styles equally important, you may assign them the same number. | | | |
|  | 1 | ( ) Transformational leader: **inspires the team** by communicating a clear direction, mobilizing people to action, based on **trust** and **motivation**.  ( ) Relational leader: acts as a **facilitator of relationships** among team members, valuing **social bonds** and **shared decision-making**.  ( ) Adaptive leader: focuses on providing **quick responses** and **disseminating information**. Mobilizes the team to face **complex problems** **without clear solutions**.  ( ) Resilient leader: demonstrates **emotional stability** and **recovery capacity** when faced with stress. Prioritizes **performance continuity** and **team support** in adverse situations. | |
| End of survey | - | Closing remarks | - |
