# Supplementary material for: Identifying informal leaders among medical residents as a basis for educational interventions
Source: BMC Med Educ. 2026 Feb 28;26:560. doi: 10.1186/s12909-026-08918-0 (PMC13059413; doi:10.1186/s12909-026-08918-0)
Supplement: Supplementary file 3 — Supplementary Material 3. [file 12909_2026_8918_MOESM3_ESM.docx]

**Supplementary file 3:** LAI and LAD scores for the full network (preceptors and residents). Note: S.D.=Standard Deviation.

| **ID** | **LAI (SD)** | **LAD (SD)** | **Quadrant Classification** |
| --- | --- | --- | --- |
| **P1** | 5.00 (0.21) | 2.44 (0.22) | Established leaders |
| **P2** | 4.39 (0.22) | 1.92 (0.19) | Aspirational leaders |
| **P3** | 4.65 (0.24) | 2.00 (0.17) | Aspirational leaders |
| **P4** | 3.47 (0.32) | 3.80 (0.35) | Humble leaders |
| **P5** | 4.79 (0.27) | 1.51 (0.20) | Aspirational leaders |
| **P6** | 4.38 (0.27) | 2.25 (0.34) | Established leaders |
| **P7** | 4.58 (0.22) | 3.31 (0.53) | Established leaders |
| **P8** | 4.06 (0.34) | 2.17 (0.26) | Latent leaders |
| **P9** | 4.57 (0.33) | 3.97 (0.35) | Established leaders |
| **P10** | 4.79 (0.27) | 2.41 (0.18) | Established leaders |
| **P11** | 4.61 (0.21) | 2.01 (0.21) | Aspirational leaders |
| **P12** | 4.57 (0.11) | 3.87 (0.30) | Established leaders |
| **P13** | 4.21 (0.17) | 2.43 (0.35) | Established leaders |
| **P14** | 4.48 (0.17) | 2.08 (0.25) | Aspirational leaders |
| **P15** | 4.42 (0.30) | 2.03 (0.20) | Aspirational leaders |
| **P16** | 4.36 (0.17) | 1.85 (0.23) | Aspirational leaders |
| **P17** | 4.80 (0.30) | 1.99 (0.22) | Aspirational leaders |
| **P18** | 5.00 (0.21) | 1.79 (0.25) | Aspirational leaders |
| **P19** | 4.14 (0.21) | 1.95 (0.28) | Latent leaders |
| **P20** | 3.52 (0.09) | 2.15 (0.26) | Latent leaders |
| **P21** | 4.51 (0.23) | 3.46 (0.43) | Established leaders |
| **P22** | 5.00 (0.21) | 1.81 (0.21) | Aspirational leaders |
| **R1.1** | 4.06 (0.34) | 2.32 (0.31) | Humble leaders |
| **R1.2** | 3.82 (0.31) | 1.63 (0.14) | Latent leaders |
| **R1.3** | 3.09 (0.32) | 1.57 (0.13) | Latent leaders |
| **R1.4** | 3.64 (0.43) | 1.96 (0.16) | Latent leaders |
| **R1.5** | 3.61 (0.27) | 1.75 (0.16) | Latent leaders |
| **R1.6** | 3.90 (0.32) | 2.74 (0.19) | Humble leaders |
| **R1.7** | 3.85 (0.40) | 1.86 (0.16) | Latent leaders |
| **R1.8** | 4.08 (0.34) | 2.71 (0.11) | Humble leaders |
| **R1.9** | 4.00 (0.17) | 1.95 (0.14) | Latent leaders |
| **R1.10** | 3.47 (0.20) | 2.22 (0.17) | Latent leaders |
| **R1.11** | 3.78 (0.45) | 2.01 (0.17) | Latent leaders |
| **R1.12** | 3.83 (0.32) | 1.76 (0.15) | Latent leaders |
| **R1.13** | 4.53 (0.27) | 2.26 (0.11) | Established leaders |
| **R1.14** | 3.49 (0.20) | 1.64 (0.18) | Latent leaders |
| **R1.15** | 4.89 (0.24) | 1.45 (0.19) | Aspirational leaders |
| **R1.16** | 4.69 (0.31) | 1.97 (0.14) | Aspirational leaders |
| **R1.17** | 3.07 (0.46) | 2.25 (0.23) | Humble leaders |
| **R1.18** | 4.10 (0.39) | 1.86 (0.24) | Latent leaders |
| **R1.19** | 4.20 (0.32) | 1.74 (0.15) | Aspirational leaders |
| **R1.20** | 4.29 (0.28) | 2.53 (0.14) | Established leaders |
| **R1.21** | 3.66 (0.28) | 2.40 (0.17) | Humble leaders |
| **R1.22** | 4.55 (0.28) | 2.29 (0.22) | Established leaders |
| **R2.1** | 4.36 (0.16) | 1.97 (0.17) | Aspirational leaders |
| **R2.2** | 4.28 (0.29) | 1.47 (0.18) | Aspirational leaders |
| **R2.3** | 3.82 (0.36) | 2.82 (0.10) | Humble leaders |
| **R2.4** | 3.59 (0.15) | 2.30 (0.19) | Humble leaders |
| **R2.5** | 4.80 (0.30) | 3.16 (0.18) | Established leaders |
| **R2.6** | 3.89 (0.20) | 1.87 (0.17) | Latent leaders |
| **R2.7** | 3.66 (0.22) | 1.65 (0.15) | Latent leaders |
| **R2.8** | 3.53 (0.23) | 2.06 (0.15) | Latent leaders |
| **R2.9** | 3.89 (0.44) | 2.30 (0.25) | Humble leaders |
| **R2.10** | 4.72 (0.19) | 1.92 (0.17) | Aspirational leaders |
| **R2.11** | 3.82 (0.36) | 2.32 (0.19) | Humble leaders |
| **R2.12** | 4.16 (0.29) | 2.64 (0.07) | Humble leaders |
| **R2.13** | 3.98 (0.37) | 2.32 (0.20) | Humble leaders |
| **R2.14** | 4.67 (0.30) | 3.84 (0.33) | Established leaders |
| **R2.15** | 4.20 (0.36) | 1.91 (0.19) | Aspirational leaders |
| **R2.16** | 4.46 (0.36) | 1.77 (0.18) | Aspirational leaders |
| **R2.17** | 3.86 (0.15) | 2.01 (0.17) | Latent leaders |
| **R2.18** | 4.43 (0.30) | 2.00 (0.19) | Aspirational leaders |
| **R2.19** | 4.43 (0.30) | 2.21 (0.15) | Aspirational leaders |
| **R2.20** | 4.79 (0.27) | 2.14 (0.17) | Aspirational leaders |
| **R2.21** | 3.69 (0.27) | 1.48 (0.19) | Latent leaders |
| **R2.22** | 4.43 (0.30) | 2.43 (0.17) | Established leaders |
| **R2.23** | 4.79 (0.27) | 2.06 (0.20) | Aspirational leaders |
| **R3.1** | 4.38 (0.27) | 2.40 (0.16) | Established leaders |
