# Supplementary material for: Identifying informal leaders among medical residents as a basis for educational interventions
Source: BMC Med Educ. 2026 Feb 28;26:560. doi: 10.1186/s12909-026-08918-0 (PMC13059413; doi:10.1186/s12909-026-08918-0)
Supplement: Supplementary file 4 — Supplementary Material 4. [file 12909_2026_8918_MOESM4_ESM.docx]

**Supplementary file 4:** Boundary sensitivity analysis: full network (preceptors and residents) vs residents-only network.

Note: Preceptors are not included in the residents-only network specification; therefore, resident-only columns are marked as NA (Not Applicable) for preceptors.

| **Code** | **LAI (preceptors and residents)** | **LAI (only residents)** | **LAD (preceptors and residents)** | **LAD (only residents)** | **Quadrant classification (preceptors and residents)** | **Quadrant classification (only residents)** | **Was there any change in quadrant classification? (Yes/No)** |
| --- | --- | --- | --- | --- | --- | --- | --- |
| **P1** | 5.00 | NA | 2.44 | NA | Established leaders | NA | NA |
| **P2** | 4.39 | NA | 1.92 | NA | Aspirational leaders | NA | NA |
| **P3** | 4.65 | NA | 2.00 | NA | Aspirational leaders | NA | NA |
| **P4** | 3.47 | NA | 3.80 | NA | Humble leaders | NA | NA |
| **P5** | 4.79 | NA | 1.51 | NA | Aspirational leaders | NA | NA |
| **P6** | 4.38 | NA | 2.25 | NA | Established leaders | NA | NA |
| **P7** | 4.58 | NA | 3.31 | NA | Established leaders | NA | NA |
| **P8** | 4.06 | NA | 2.17 | NA | Latent leaders | NA | NA |
| **P9** | 4.57 | NA | 3.97 | NA | Established leaders | NA | NA |
| **P10** | 4.79 | NA | 2.41 | NA | Established leaders | NA | NA |
| **P11** | 4.61 | NA | 2.01 | NA | Aspirational leaders | NA | NA |
| **P12** | 4.57 | NA | 3.87 | NA | Established leaders | NA | NA |
| **P13** | 4.21 | NA | 2.43 | NA | Established leaders | NA | NA |
| **P14** | 4.48 | NA | 2.08 | NA | Aspirational leaders | NA | NA |
| **P15** | 4.42 | NA | 2.03 | NA | Aspirational leaders | NA | NA |
| **P16** | 4.36 | NA | 1.85 | NA | Aspirational leaders | NA | NA |
| **P17** | 4.80 | NA | 1.99 | NA | Aspirational leaders | NA | NA |
| **P18** | 5.00 | NA | 1.79 | NA | Aspirational leaders | NA | NA |
| **P19** | 4.14 | NA | 1.95 | NA | Latent leaders | NA | NA |
| **P20** | 3.52 | NA | 2.15 | NA | Latent leaders | NA | NA |
| **P21** | 4.51 | NA | 3.46 | NA | Established leaders | NA | NA |
| **P22** | 5.00 | NA | 1.81 | NA | Aspirational leaders | NA | NA |
| **R1.1** | 4.06 | 4.06 | 2.32 | 2.55 | Humble leaders | Humble leaders | No |
| **R1.2** | 3.82 | 3.82 | 1.63 | 1.94 | Latent leaders | Latent leaders | No |
| **R1.3** | 3.09 | 3.09 | 1.57 | 2.09 | Latent leaders | Latent leaders | No |
| **R1.4** | 3.64 | 3.64 | 1.96 | 2.35 | Latent leaders | Latent leaders | No |
| **R1.5** | 3.61 | 3.61 | 1.75 | 2.32 | Latent leaders | Latent leaders | No |
| **R1.6** | 3.90 | 3.90 | 2.74 | 3.00 | Humble leaders | Humble leaders | No |
| **R1.7** | 3.85 | 3.85 | 1.86 | 2.20 | Latent leaders | Latent leaders | No |
| **R1.8** | 4.08 | 4.08 | 2.71 | 3.00 | Humble leaders | Established leaders | Yes |
| **R1.9** | 4.00 | 4.00 | 1.95 | 2.41 | Latent leaders | Latent leaders | No |
| **R1.10** | 3.47 | 3.47 | 2.22 | 2.26 | Latent leaders | Latent leaders | No |
| **R1.11** | 3.78 | 3.78 | 2.01 | 2.44 | Latent leaders | Latent leaders | No |
| **R1.12** | 3.83 | 3.83 | 1.76 | 2.28 | Latent leaders | Latent leaders | No |
| **R1.13** | 4.53 | 4.53 | 2.26 | 2.69 | Established leaders | Established leaders | No |
| **R1.14** | 3.49 | 3.49 | 1.64 | 2.13 | Latent leaders | Latent leaders | No |
| **R1.15** | 4.89 | 4.89 | 1.45 | 1.55 | Aspirational leaders | Aspirational leaders | No |
| **R1.16** | 4.69 | 4.69 | 1.97 | 2.56 | Aspirational leaders | Established leaders | Yes |
| **R1.17** | 3.07 | 3.07 | 2.25 | 2.43 | Humble leaders | Latent leaders | Yes |
| **R1.18** | 4.10 | 4.10 | 1.86 | 2.02 | Latent leaders | Aspirational leaders | Yes |
| **R1.19** | 4.20 | 4.20 | 1.74 | 2.15 | Latent leaders | Latent leaders | No |
| **R1.20** | 4.29 | 4.29 | 2.53 | 2.67 | Established leaders | Established leaders | No |
| **R1.21** | 3.66 | 3.66 | 2.40 | 2.65 | Humble leaders | Humble leaders | No |
| **R1.22** | 4.55 | 4.55 | 2.29 | 2.64 | Established leaders | Established leaders | No |
| **R2.1** | 4.36 | 4.36 | 1.97 | 2.40 | Aspirational leaders | Aspirational leaders | No |
| **R2.2** | 4.28 | 4.28 | 1.47 | 1.78 | Aspirational leaders | Aspirational leaders | No |
| **R2.3** | 3.82 | 3.82 | 2.82 | 4.02 | Humble leaders | Humble leaders | No |
| **R2.4** | 3.59 | 3.59 | 2.30 | 2.67 | Humble leaders | Humble leaders | No |
| **R2.5** | 4.80 | 4.80 | 3.16 | 3.39 | Established leaders | Established leaders | No |
| **R2.6** | 3.89 | 3.89 | 1.87 | 2.48 | Latent leaders | Latent leaders | No |
| **R2.7** | 3.66 | 3.66 | 1.65 | 2.12 | Latent leaders | Latent leaders | No |
| **R2.8** | 3.53 | 3.53 | 2.06 | 2.67 | Latent leaders | Humble leaders | Yes |
| **R2.9** | 3.89 | 3.89 | 2.30 | 2.57 | Humble leaders | Humble leaders | No |
| **R2.10** | 4.72 | 4.72 | 1.92 | 2.40 | Aspirational leaders | Aspirational leaders | No |
| **R2.11** | 3.82 | 3.82 | 2.32 | 2.98 | Humble leaders | Humble leaders | No |
| **R2.12** | 4.16 | 4.16 | 2.64 | 3.24 | Humble leaders | Established leaders | Yes |
| **R2.13** | 3.98 | 3.98 | 2.32 | 2.53 | Humble leaders | Latent leaders | Yes |
| **R2.14** | 4.67 | 4.67 | 3.84 | 4.63 | Established leaders | Established leaders | No |
| **R2.15** | 4.20 | 4.20 | 1.91 | 2.48 | Latent leaders | Latent leaders | No |
| **R2.16** | 4.46 | 4.46 | 1.77 | 2.25 | Aspirational leaders | Aspirational leaders | No |
| **R2.17** | 3.86 | 3.86 | 2.01 | 2.63 | Latent leaders | Humble leaders | Yes |
| **R2.18** | 4.43 | 4.43 | 2.00 | 2.35 | Aspirational leaders | Aspirational leaders | No |
| **R2.19** | 4.43 | 4.43 | 2.21 | 2.64 | Aspirational leaders | Established leaders | Yes |
| **R2.20** | 4.79 | 4.79 | 2.14 | 2.56 | Aspirational leaders | Established leaders | Yes |
| **R2.21** | 3.69 | 3.69 | 1.48 | 1.89 | Latent leaders | Latent leaders | No |
| **R2.22** | 4.43 | 4.43 | 2.43 | 2.89 | Established leaders | Established leaders | No |
| **R2.23** | 4.79 | 4.79 | 2.06 | 2.52 | Aspirational leaders | Aspirational leaders | No |
| **R3.1** | 4.38 | 4.38 | 2.40 | 2.44 | Established leaders | Aspirational leaders | Yes |
